# Fluorescent Labeling of Antibody Fragments Using Split GFP

**DOI:** 10.1371/journal.pone.0025727

**Published:** 2011-10-05

**Authors:** Fortunato Ferrara, Pawel Listwan, Geoffrey S. Waldo, Andrew R. M. Bradbury

**Affiliations:** Bioscience Division, Los Alamos National Laboratory, Los Alamos, New Mexico, United States of America; Cardiff University, United Kingdom

## Abstract

Antibody fragments are easily isolated from *in vitro* selection systems, such as phage and yeast display. Lacking the Fc portion of the antibody, they are usually labeled using small peptide tags recognized by antibodies. In this paper we present an efficient method to fluorescently label single chain Fvs (scFvs) using the split green fluorescent protein (GFP) system. A 13 amino acid tag, derived from the last beta strand of GFP (termed GFP11), is fused to the C terminus of the scFv. This tag has been engineered to be non-perturbing, and we were able to show that it exerted no effect on scFv expression or functionality when compared to a scFv without the GFP11 tag. Effective functional fluorescent labeling is demonstrated in a number of different assays, including fluorescence linked immunosorbant assays, flow cytometry and yeast display. Furthermore, we were able to show that this split GFP system can be used to determine the concentration of scFv in crude samples, as well an estimate of antibody affinity, without the need for antibody purification. We anticipate this system will be of widespread interest in antibody engineering and *in vitro* display systems.

## Introduction

Monoclonal antibodies (mAbs) have long been used for biological and medical research, as well as *in vitro* diagnostics. However, the derivation of mAbs is relatively expensive and time consuming. Over the past decade, an alternative and effective way to obtain monoclonal antibodies has been the use of recombinant antibody display libraries from which antibodies of interest can be selected and expressed in *E. coli* [1]. Filamentous phage display [2] is the most widely distributed display platform, with yeast [3] and ribosome display [4] also frequently used. The commonest antibody formats used are single chain Fvs (scFvs) [5] and Fabs. scFvs are recombinant constructs in which the variable domains of the heavy and light chains are covalently linked together by a flexible linker [5], whereas Fabs were originally described by the digestion of antibody with papain [6], and consist of two protein fragments, VH-CH1, and VL-CL that can be expressed separately in the *E. coli* periplasm where they assemble together [7]. Full-length antibodies include the Fc region, which, from a reagent point of view, can be considered to be a large tag recognized by secondary antibodies or bacterial superantigens, such as protein A or G. Antibody fragments, in contrast, are usually modified by the fusion of in-frame tags to the antibody fragment. The commonest format includes a short peptide tag (e.g. myc [8] or SV5 [9]) recognized by a monoclonal antibody, and a histidine tag that can be used for purification by immobilized metal affinity chromatography [10]. Detection of antibody fragments using peptide tags generally requires an extra step when compared to the detection of full-length antibodies, since anti-peptide and subsequent labeled secondary antibodies are usually required. Given the recombinant nature of antibody fragments selected from phage display libraries, it was initially thought it would be relatively trivial to fuse effector domains directly to antibody fragments. Direct fusion to alkaline phosphatase has been extremely successful [11]–[14], with scFv-AP fusions being relatively well expressed and stable, allowing direct enzyme linked immunosorbant assays (ELISAs) to be carried out without the need for additional reagents. In a similar manner, covalent fusion to green fluorescent protein (GFP) or similar proteins should allow fluorescent labeling of scFvs. This would also provide the significant advantage that each scFv would be labeled with only one fluorophore, potentially allowing scFv quantification by fluorescence. Although a number of publications [15]–[17] deal with the creation of such fluorescent antibody fragments, the yields have been very disappointing, mainly due to the fact that scFvs contain disulfide bonds that require oxidizing environments (such as eukaryotic secretory pathways, or the bacterial periplasm) for correct folding, while GFP folds in the reducing cytoplasm and not in the periplasm [18]. This is in contrast to alkaline phosphatase, which folds in the same oxidizing environments as antibody fragments, explaining the greater success attained with this fusion.

Recently, a number of reports [19]–[21] have described split GFP assays, in which self-complementing fragments of GFP are non-fluorescent individually, but recreate functional GFP when present together. One of these systems, in particular [19], is based on a small 13 amino acid fragment corresponding to the 11^th^ strand of GFP (GFP11) that was evolved to have minimal effect on the function or solubility of the protein to which it is fused. This peptide can restore fluorescence to an evolved version of the first 10 strands of GFP (GFP1-10) with which it interacts almost irreversibly. A potential advantage of tagging with the small split GFP is that the protein of interest can complete folding without the interference of a full-length GFP attached. Furthermore, the small split GFP tag is less likely to interfere with normal transport to the periplasm.

In this study, we described the genetic fusion of the GFP11 peptide to the well-characterized anti-chicken-lysozyme scFv, D1.3, and its expression and functionality after expression in *E. coli*. The utility and detectability of the recombinant antibody was evaluated in a fluorescence linked immunosorbant assay (FLISA) [17], [22], [23], an immunosorbant assay that uses fluorescence rather than enzymatic activity for detection, in yeast display, and in a multiplex flow cytometry assay for high-throughput purposes.

## Materials and Methods

### DNA constructs

**Figure 1:**
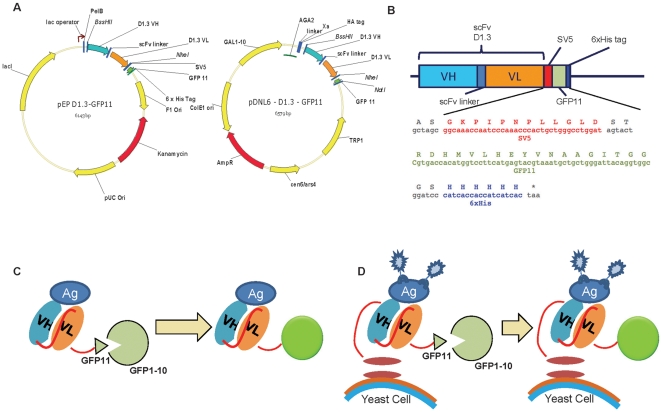
Split-GFP labeling of scFvs. **A. GFP complementation vectors.** The scFv-D1.3-GFP11 construct was cloned into a pEP based vector (left) or a yeast display vector (right). The GFP11 peptide is cloned at the C-terminus of the scFv in order not to interfere with the scFv/antigen-binding activity. **B. Linear representation of the scFv-GFP11 molecule.** The sequence of the SV5 tag, followed by the GFP11 sequence and the six histidine tag (6xHis tag) found at the C terminus of the scFv is shown. **C. Split GFP fragment complementation.** The scFv is fused to the small GFP fragment (strand 11, residues 215-230). The complementary GFP fragment (1-10, residues 1-214) is expressed separately. Neither fragment alone is fluorescent. When mixed, the small and large GFP fragments spontaneously associate, resulting in reconstitution of the fluorophore and fluorescence. **D. Split GFP fragment complementation in scFv yeast display.** The scFv in fusion with the GFP11 strand is displayed on yeast cells. It is still able to bind the specific antigen and fluorescence is restored when yeast cells are incubated with the GFP1-10 fragment.

The GFP11 Fragment was created by annealing the two oligonucleotides GFP11-rev (P – GAT CCG CCA CCT GTA ATC CCA GCA GCA TTT ACG TAC TCA TGA AGG ACC ATG TGG TCA CGA GT) and GFP11-for (P – ACT CGT GAC CAC ATG GTC CTT CAT GAG TAC GTA AAT GCT GCT GGG ATT ACA GGT GGC G). This creates the GFP11 fragment with restriction site ends (ScaI and BamHI) compatible with the pEP-D1.3 vector [24] (a pET based scFv expression vector, in which an E coil [25] is in fusion with the scFv) (Figure 1a). The final construct consists of the D1.3 scFv fused to GFP11and a His tag at the C terminus (Figure 1b–c), with the E-coil removed.

In order to generate the yeast display vector, pDNL6-GFP11, the D1.3-GFP11 fragment was PCR amplified using a modified GFP11-forward primer that replaces the BamHI restriction site with NotI. Purified fragments were digested with BssHII and NotI and cloned into pDNL6, a yeast display vector based on pPNL6 [26] which contains BssHII and NheI as cloning sites in order to be compatible with our phage display vector, pDAN5 [27] (Figure 1a).

### Antigen preparation

Neutravidin (Pierce Inc.), Lysozyme and Myoglobin, were purchased (SIGMA Aldrich), while IgE receptor was donated by Graham Mackay, University of Melbourne. All antigens were biotinylated using the sulfo NHS biotinylation system (Pierce Inc.) according to the manufacturer's instructions. The purified anti-SV5 antibody was conjugated with alkaline phosphate enzyme (AP) using the Lightning-LinkTM AP Conjugation Kit (Innova Biosciences) according to the manufacturer's instructions.

### Expression and purification of scFv-D1.3-GFP11 and GFP1-10

pEP-D1.3-GFP11 and pEP-D1.3-Ecoil DNA were transformed into BL21 (DE3) (Novagen). Bacteria were grown in 50 ml of auto-induction media [28] at 25°C overnight to produce the recombinant protein.

Bacteria were harvested and resuspended in 10 ml PBS. Lysis was performed by means of a pressure press (EmulsiFlex–C5, Avestin Inc.). The bacterial debris was pelleted by centrifugation, and the protein purified from the supernatant using the C-terminal His-tag and Ni-NTA agarose resin (Qiagen Inc.) according to the manufacturer's instructions. Purified protein was dialyzed against PBS and stored at −20°C.

Crude protein extract or bacterial culture supernatant was used. After expression in 1 ml of selective auto-induction media, cells were harvested, the supernatant was recovered, and the pellet resuspended in 1/10 volume of Pop culture (Novagen) solution. Lysis was performed for 25 minutes at room temperature (RT), with shaking, and the suspension used after centrifugation without further protein purification. GFP 1-10 was prepared as previously described [19], [29], [30]; 75 mg of inclusion body GFP1-10 was unfolded in 1 ml 9 M urea, and refolded in 20 ml TNG buffer (100 mM Tris HCl pH 7.5, 150 mM NaCl, 10% v/v glycerol) prior to use. Quantification was carried out by comparing the intensity of purified scFv-D1.3-GFP11 to known concentrations of BSA standards on poly-acrylamide gel stained with gel code (Pierce Inc.).

### Complementation assay

A 96-well microplate was first blocked with a solution of 0.5% bovine serum albumin (BSA) in PBS for 30 minutes. Different concentrations of purified scFv-D1.3-GFP11 were prepared (75, 150, 300 pmol) in a final volume of 100 µl. A large molar excess (800 pmol) of GFP 1-10 in 180 µl was added and complementation carried out. Crude Pop-culture *E.coli* lysate was also tested using two fold serial dilutions in a final volume of 50 µl, incubated with a similar large molar excess of GFP1-10.

Fluorescence (λ_exc_ = 488 nm/λ_em_  = 530 nm) was monitored using a Tecan Infinite M200 reader measuring at 3 minutes intervals for 20 hours for the purified protein and 10 hours for the crude extracts at room temperature (RT).

### Calibration curve

To correlate the fluorescent signal with the D1.3-GFP11 protein quantity present in the crude bacteria extract, two calibration curves were generated by measuring the fluorescence after split-GFP complementation of a control antigen, sulfite-reductase (SR-GFP11), which we have used extensively [29] and of purified scFv-D1.3-GFP11, both of known concentration (range 1.5–160 pmol) and molecular weight. Forty µl of purified protein solutions or total cell extract were mixed with 190 µl of 800 pmol GFP 1-10 protein solution. The samples were incubated over night (ON) at 4°C. Fluorescence was measured using the Beckman Coulter DTX-880 Multimode Detector. The quantity of scFv-D1.3-GFP11 in the crude bacteria extract was calculated using the equations in Canbantou et al [31].

### Enzyme linked immunosorbant assay (ELISA)

100 µl of neutravidin solution (10 µg/ml concentration) were added to each well of an ELISA plate (Nunc 442404) and incubated at 37°C for 1 hour in order to coat the plate. The wells were washed twice with PBSLT (PBS, Tween 20 0.05%), then blocked with 200 µl of 1% BSA in PBS for 1 hour at RT. The blocking solution was washed away with PBSLT. 100 µl of biotinylated antigen (10 µg/ml) were added to each well and incubated for 30 minutes at RT. After extended washing with PBSLT, 100 µl of different concentrations of the scFv-D1.3-GFP11 or D1.3-Ecoil purified protein (range 0.6 – 300 nM) were added and incubated for 1 hour at room temperature. Unbound proteins were removed by washing three times with PBST (PBS, Tween 20 0.5%) and three times with PBSLT. 100 µl of anti-SV5 AP (1µg/ml) solution was added and incubated for 1 hour at RT. Washing steps were repeated (3xPBST, 3xPBSLT), and alkaline phosphatase activity detected using PNPP solution (Sigma) with absorbance read at 405 nm.

Equilibrium binding titration curve for the complemented D1.3-GFP11 to determine the affinity constant Kd of scFv-D1.3-GFP11 was obtained by nonlinear regression using PRISM software (version 5.0 Graph Pad).

### Fluorescence linked immunosorbant assay (FLISA)

Neutravidin coating and antigen binding were performed following the ELISA protocol, except that black (Nunc 43711) ELISA plates were used as previously described [23]. After antigen coating, 50 µl of different concentrations of the scFv-D1.3-GFP11 purified protein (ranging from 0.6 to 300 nM),, were added and incubated for 1 hour at RT. Unbound proteins were removed by washing three times with PBST and three times PBSLT. 90 µl of 800 pmol GFP1-10 were added to each well and fluorescence was read after 4 hours at RT.

Equilibrium binding titration curve to determine the affinity constant Kd of scFv-D1.3-GFP11 was obtained by using nonlinear regression running PRISM software (version 5.0 Graph Pad).

### Yeast display

The scFv-D1.3-GFP11 construct was cloned into the yeast display vector pDNL6, to create pDNL6-GFP11, and transformed into *S. cerevisiae* EBY100 according to the Yeast transformation kit manual (Sigma). A single clone was then grown in SD-CAA selective media at 30°C. Cells were diluted to 0.5 OD_600_ in induction media SG/R + CAA and incubated at 20°C with shaking (around 20 hours). 2×10^6^ yeast induced cells were washed with 1 ml of washing buffer (PBS, BSA 0.5%, EDTA 20mM) and resuspended in 100 µl containing: i) 1 µl of anti-SV5 mAb (1 mg/ml) plus 100 nM biotinylated antigen together; ii) anti-SV5 alone; or iii) biotinylated antigen alone. After 30 minutes of incubation at RT followed by 5 minutes on ice, cells were spun down and washed twice with 500 µl of ice-cold washing buffer.

Cells were resuspended in 100 µl of a 1/200 dilution of secondary reagents: streptavidin-Alexa633 for antigen detection and anti-mouse-PE conjugated for anti SV5-scFv detection. The cells were incubated for 30 minutes in ice, harvested and washed twice with cold buffer. Cells were then resuspended in 1 ml of PBS or GFP1-10 solution (3.75 mg/ml, 4 µM).

Florescence data were collected using BD FACSAria flow Cytometer and analyzed using DIVA software. The yeast population was gated and the mean fluorescent values for PE (corresponding to scFv expression), APC (corresponding to antigen binding) and FITC (corresponding to complementation of scFv-D1.3-GFP11 with GFP10) were recorded using appropriate excitation lasers and emission filters.

### Multiplexed Flow Cytometry

Three different color-coded, carboxylated microsphere sets were used (Luminex, xMAP 135, 129, 159). Each individual set was coupled with Neutravidin, coupling was quantified [24], and biotinylated antigens were respectively bound: lysozyme to beads 135, myoglobin to beads 129 and IgE receptor to beads 159.

16 µl (1.6×10^5^ microspheres) of each bead set were incubated with 100 µl of different concentrations of purified protein (ranging from 0.38 to 380 pmol) or with POP culture extract. The solutions were then incubated with a molar excess of GFP1-10 for two hours at RT, and the mean fluorescence data of each bead set was collected using the high-throughput analysis feature of a Becton Dickinson LSRII Flow Cytometer and analyzed using the DIVA software package. The bead multiplex was separated into gates using the setting previously described [24] and the mean fluorescent value of each gate was recorded following excitation using 488 nm laser through FITC detectors. Equilibrium binding titration curves to determine the affinity constant Kd of scFv-D1.3-GFP11 was obtained by nonlinear regression using PRISM software (version 5.0 Graph Pad).

## Results

The small 13-amino-acid 11^th^ strand of GFP (GFP11) was generated by PCR, annealing two phosphorylated oligonucleotides with appropriate restriction sites and cloning at the C-terminus of the D1.3 scFv gene in the pEP vector (Fig. 1). The His-tag at the C-terminus of GFP11 was used for purification by immobilized metal chromatography. The yield of scFv-D1.3-GFP11 was up to 5 mg per liter of bacterial culture.

### Binding activity comparison

To determine whether fusion to the GFP11 peptide interferes with the expression and binding activity of the scFv, the scFv-D1.3-GFP11 molecule was compared to an essentially identical D1.3 scFv in fusion with the E-coil peptide [25], and lacking the GFP11 tag.

**Figure 2:**
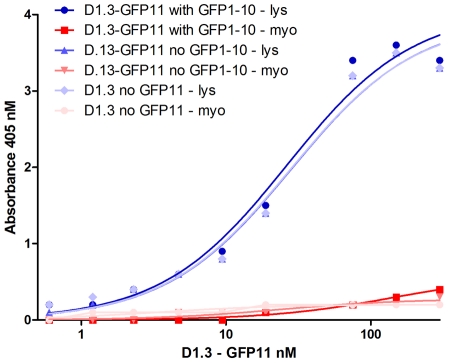
Comparison of antigen binding activity assessed by ELISA. The antigen binding activity was analyzed by ELISA using different concentrations of scFv-D1.3 and scFv-D1.3-GFP11 scFvs (ranging from 0.6 to 300 nM). The scFv-D1.3-GFP11 construct showed good binding activity for lysozyme (lys) even after incubation with the GFP1-10 complementing protein, indicating the absence of steric hindrance between the restored GFP protein and the anti-SV5 antibody used for detection. Myoglobin (myo) was used as negative control.

Equal quantities of D1.3-E-coil and scFv-D1.3-GFP11 were used in an ELISA assay. The antibody antigen interaction was detected using SV5 antibodies and the scFv-D1.3-GFP11 was used with and without GFP1-10 incubation. The results were very similar for both constructs, at different concentrations, whether GFP1-10 was added or not, indicating that the GFP11 tag does not sterically hinder binding to the SV5 tag, even when the GFP molecule is fully complemented at the C-terminus of the scFv (Figure 2).

### Complementation assay

**Figure 3:**
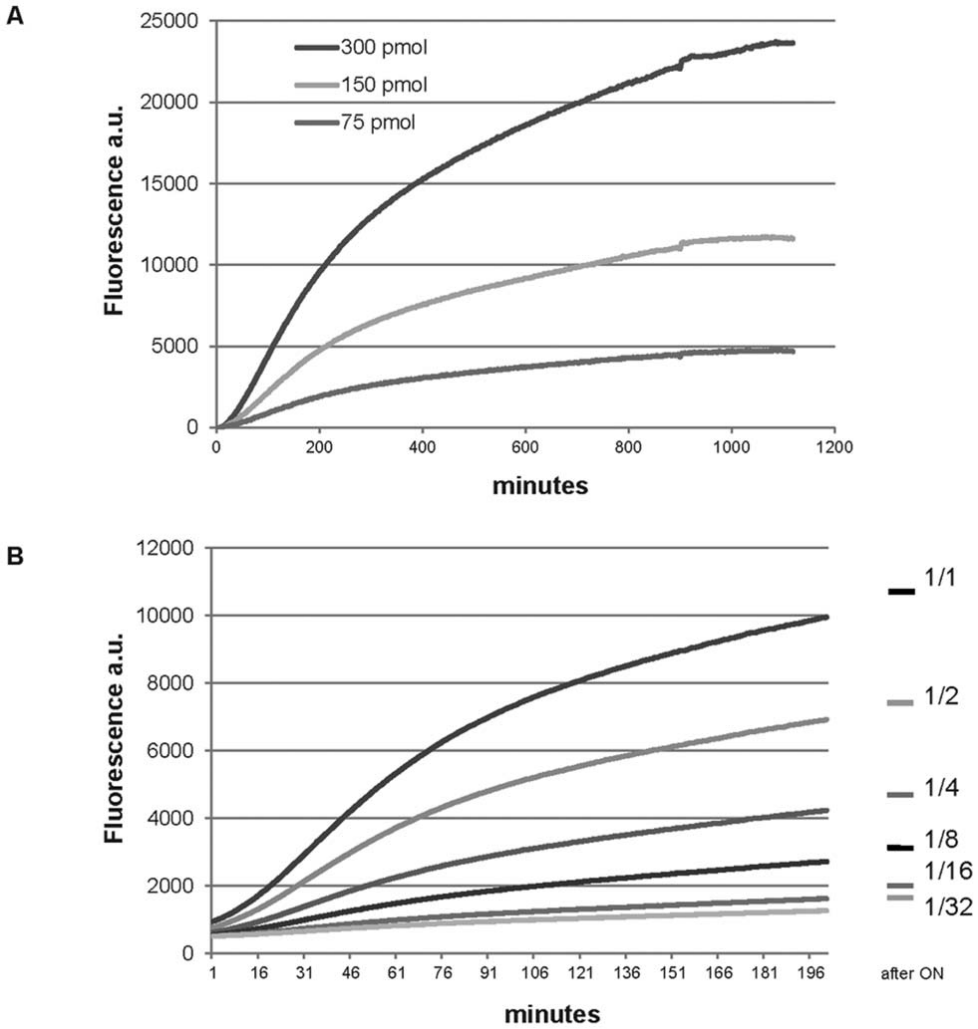
Progress curves analysis of the complemented fluorescence. **A. Progress curves of complemented fluorescence using purified protein.** The increasing fluorescence over time was determined (a.u arbitrary units) for the complementation of scFv-D1.3-GFP11 samples at different concentrations (300, 150, 75 pmol) incubated with an excess of GFP1-10 (800 pmol). Fluorescence (λ_exc_ = 488 nm/λ_em_ = 530 nm) was monitored at 3 minutes intervals for 20 h at RT. **B. Progress curves of complemented fluorescence using serial dilutions of crude bacteria extract.** A similar trend of increasing fluorescence was measured (a.u arbitrary units) directly using serial dilution of crude bacterial culture extract, albeit at lower fluorescent values. Fluorescence (λ_exc_ = 488 nm/λ_em_ = 530 nm) was monitored at 3 minutes intervals for 10 hours at RT.

In order to determine how effectively split GFP could be used to label a scFv molecule, we tested the complementation between serial dilutions of purified scFv-D1.3-GFP11 protein (75, 150, 300 pmol) and a molar excess of GFP1-10 (800 pmol). We measured the progression of fluorescence, indicating complementation, in a microtiter plate (Figure 3a). The fluorescent signal could be easily detected when illuminated at 488 nm within an hour after the addition of the complementary fragment GFP1-10 at room temperature with the fluorescence levels directly related to the different concentrations of scFv-D1.3-GFP11. In the absence of GFP1-10, there was no fluorescence. As protein purification is a time consuming process, we also carried out the assay using serial dilutions of a crude bacterial lysate. The curves obtained showed a significant increase in fluorescence after incubation with the GFP1-10 fragment, directly related to the scFv-D1.3-GFP11 dilution (Figure 3b).

### Quantification of scFv-D1.3-GFP11 using calibration curves

**Figure 4:**
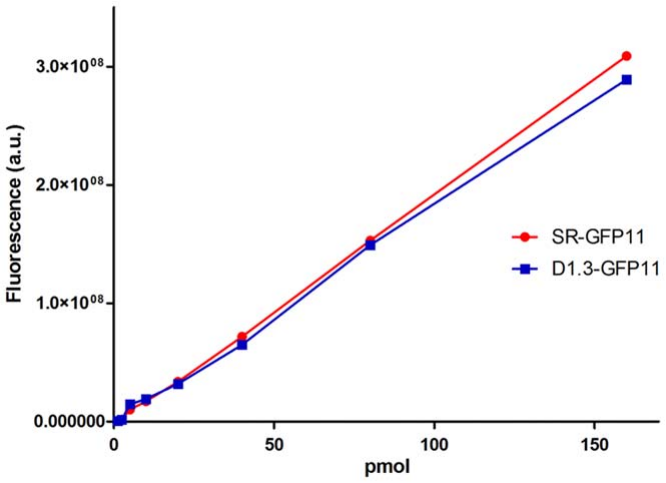
GFP1-10 standard curves to evaluate protein concentration. A GFP11 standard plot was constructed using increasing amounts of sulfite reductase GFP11 (SR-GFP11) and scFv-D1.3-GFP11 (ranging from 1.5 to 160 pmol) in a molar excess of GFP1-10. The quantity of GFP11 tagged scFv in the cell extract was calculated according to the equations presented in Canbantou et al. [31]. In all cases fluorescence (a.u. arbitrary units) was measured after overnight incubation at 4°C.

In previous papers, GFP complementation using the GFP11 tag has been used to quantify proteins in E. coli [29] and eukaryotic cell extracts [32]. We assessed the ability to use the GFP11 tag to quantify scFvs in crude cell extracts using two standard curves prepared with complemented dilutions of sulfite-reductase GFP11 and purified scFv-D1.3-GFP11. Sulfite-reductase is the protein standard that we have previously used to calibrate GFP11 tagged proteins [30], [31], [33]. As shown in figure 4, there is a direct linear relationship between fluorescence and the concentration of SR-GFP11 and scFv-D1.3-GFP11, resulting in two almost superimposable calibration curves. By measuring the fluorescence of a complemented sample of crude scFv-D1.3-GFP11 obtained from bacterial expression in deep well microtiter plates, and using the equation presented in Cabantous et al. [31], we were able to calculate the expression level of complemented scFv-D1.3-GFP11 under these conditions to be 5.6 µg/ml. This is approximately ten fold less than the amount of protein we routinely obtain from expression in large flasks, where aeration is signficantly better, and purification is probably more efficient.

### Fluorescence linked immunosorbant assay (FLISA)

**Figure 5:**
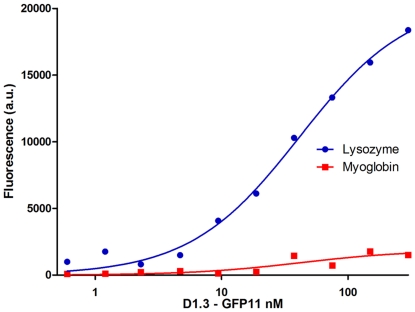
FLISA results at different complemented scFv-GFP11 concentrations. scFv-D1.3-GFP11 was used in a FLISA assay with binding between the scFv and its specific antigen assessed by complemented fluorescence. No signal was detected for the negative control myoglobin.

Having shown that GFP11 was functional when fused to the C terminus of an scFv, we tested the antigen binding properties of the fluorescent scFv using a fluorescent linked immunosorbant assay [23], which exploited the fluorescence of the complemented GFP11. We have previously shown that FLISA is an effective and more rapid alternative to the use of ELISA to screen scFvs, when those scFvs are made fluorescent [23]. In particular, it is a single step assay that does not require additional incubations, beyond the addition of the fluorescently labeled antibody. Figure 5 shows that the assay has a very high signal to noise ratio when scFvs are fluorescently labeled using the split GFP system. An approximation of the affinity for D1.3-GFP11 was calculated by nonlinear regression using the PRISM software package (version 5.0 Graph Pad) as 41.4 nM. This value is similar to that obtained when affinity was estimated by ELISA: Kd 25.2 nM. This falls within the range (3-500 nM) of most estimates of D1.3 affinity calculated using a number of different methods [34], [37]–[41], indicating the further utility of GFP complementation in affinity determination. Although the absence of fluorescence in the non-complemented GFP1-10, ensures that only the scFv is fluorescent, this does not avoid a small degree of non-specific binding of the complemented scFv to the negative control antigen (myoglobin), and will depend upon the specific binding properties of each individual scFv.

### Yeast display

**Figure 6:**
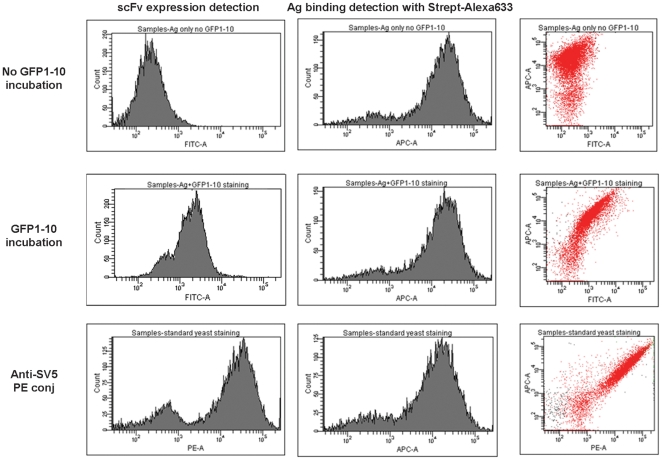
Yeast display of scFv-D1.3-GFP11. The scFv-D1.3-GFP11 containing both the GFP11 peptide as well as the peptide epitope recognized by SV5 was displayed on the surface of yeast cells. Biotinylated lysozyme was detected using Alexa 633 labeled streptavidin, and display levels were assessed using either GFP1-10 or phycoerythrin labeled SV5. Labeling by GFP complementation was essentially as effective in normalizing display levels as the use of PE labeled SV5.

Yeast display [3] is a technique in which antibodies are displayed on the surface of yeast particles and selected on the basis of their ability to bind to fluorescently labeled antigens. Clones showing increased binding can then be sorted from those showing less binding. In order to ensure that selected clones have higher affinity and not merely increased display levels, binding is usually normalized using an antibody recognizing a peptide tag at the C terminus of the antibody. Given the results we obtained above, it became clear that we may be able to use GFP complementation to carry out normalization of display rather than using anti-tag antibodies. We created yeast display constructs in which scFv-D1.3 was displayed on the surface, containing both the GFP11 peptide as well as the peptide epitope recognized by SV5 (Figure 1d). Biotinylated lysozyme was detected using Alexa 633 labeled streptavidin, and display levels were assessed using either GFP1-10 or phycoerythrin labeled SV5. The results in figure 6 show that labeling by GFP complementation was essentially as effective in normalizing display levels as the use of PE labeled SV5. The only major difference between the two labeling methods was that labeling was more intense with Phycoerythrin labeled SV5, reflecting the fact that each Phycoerythrin contains 34 fluorochromes while each GFP contains a single fluor [35], [36]. This demonstrates the potential utility of using split GFP to label antibodies in this application as well.

### Multiplex

In a recent paper we demonstrated the advantages of multiplex flow cytometry in the screening of scFvs following selection [24]. In that paper, scFvs were labeled using a fluorescent K coil that bound to an E coil appended to the C terminus of the scFv. Although we were able to obtain high signal to noise using this labeling system, one problem is that the fluorescently labeled K coil is fluorescent even when not bound to scFv, and under some circumstances demonstrated some binding activity of its own. One conceptual advantage of the use of split GFP to label scFvs is that only the complemented GFP1-10 is fluorescent. Since the only source of GFP11 is that fused to the scFv, this should improve signal to noise, by eliminating extraneous fluorescence.

**Figure 7:**
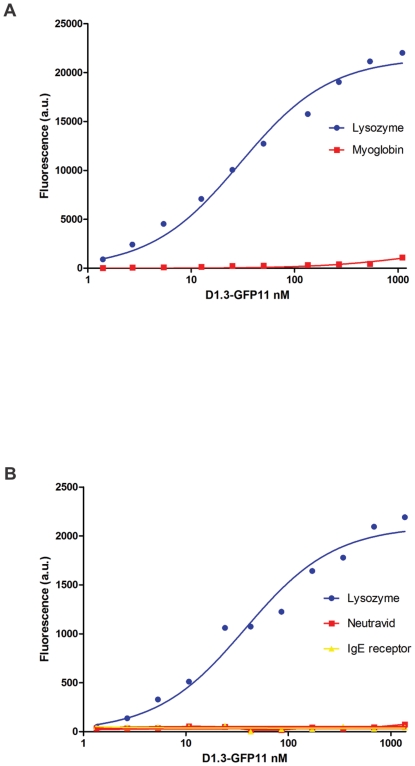
Affinity determination. **A. Affinity determination by multiplexed assay using purified protein.** After 2 hours of complementation with the GFP1-10 fragment, the scFv-D1.3-GFP11 shows specific binding to its antigen, chicken lysozyme, and no binding activity to the negative control, myoglobin. Fluorescence values are plotted versus the different concentrations of purified scFv-D1.3-GFP11 giving a Kd value of 29±4 nM. Data are fit with a nonlinear least squares regression and are visualized with the concentration values on a log scale. **B.** **Affinity determination by multiplexed assay using serial dilution of bacterial lysate (POP culture extract) obtained from induced cells.** Unpurified scFv-D1.3-GFP11 obtained from induced bacteria shows specific binding to lysozyme, but not to myoglobin or IgE receptor. Fluorescence values are plotted versus the different concentrations of scFv-D1.3-GFP11 present in crude bacteria extract calculated by reference to the GFP complementation standard (figure 4), resulting in a Kd value of 38±7 nM. Data are fit with a nonlinear least squares regression and are plotted with the concentration values on a log scale.

Functional analysis of the scFv-D1.3-GFP11 construct was first performed using *purified* protein, to correlate the fluorescent signal with a precise amount of scFv. In this first experiment two sets of beads were used, one coated with the specific antigen lysozyme and one with myoglobin as a negative control. The fluorescence values obtained showed excellent correlation between the concentration of the fluorescently labeled scFv and the signal (Figure 7a), even down to relatively low concentrations (5 nM). Furthermore, titration of the GFP complemented scFv allowed us to calculate the approximate affinity by nonlinear regression (using PRISM), as 29±4 nM, similar to the values obtained above by ELISA and FLISA.

In general, the most time-consuming aspect of affinity determination is purification of the scFv and the determination of concentration. Given that we were able to obtain the concentration of scFv-D1.3-GFP11 in the bacterial lysate using the standard curve in figure 4 (5.6 µg/ml) we also attempted to determine the affinity of the D1.3 scFv using crude samples, rather than the purified samples described above. Complementation was carried out with a molar excess of GFP1-10 added to serial dilutions of POP culture (EMD) extract. The results clearly demonstrate the ability to detect antibody bound to specific antigen (lysozyme) and not to the negative controls (myoglobin and IgER) (Figure 7b), without the need for purification. This allowed us to approximate the affinity of D1.3 scFv in the crude extract to 38±7 nM, similar to the value of 29 nM previously obtained using purified scFv (Figure 7a). This indicates the value of the split GFP system to calculate approximate scFv affinities that can be used in screening, particularly in high throughput multiplex processes in which protein purification can be avoided.

## Discussion

Fluorescently labeled antibodies can be extremely useful research and diagnostic tools. Full-length antibodies have been chemically linked to different fluorophores, including FITC, TRITC CY-3 and CY-5. This sometimes leads to reduced binding to target antigen, especially when conjugation occurs within the antigen binding site [42]. It is also difficult to control the number of labels incorporated, with considerable variability from antibody to antibody. Direct conjugation of scFv molecules to fluorescent dyes (e.g. CY-5) has also been carried out [43], although this requires prior scFv purification. Many reports describing direct fusion of GFP to scFvs have been published [15], [16], [18], [44]–[46]. However conventional scFv are prone to aggregation and the fusion between scFv and GFP augments these scFv aggregation tendencies. Furthermore, careful reading of these reports reveals that the yields for scFv-GFP fusions are extremely low, due to the incompatible folding requirements of the two moieties: antibody fragments prefer oxidizing environments, while fluorescent proteins prefer reducing environments. One exception to this general trend has been the fusion of GFP to scFvs that can fold and are functional in the cytoplasm [46]. More recently recognition molecules based on GFP with antibody-like binding proprieties have been described [47], representing an interesting new approach to the creation of fluorescent affinity reagents.

The split GFP system is able to overcome problems of expression and folding while providing fluorescent labeling characteristics to the scFv. As the GFP11 tag was evolved to be minimally intrusive [19], its fusion to the C terminus of proteins, including scFvs, has little effect on expression, solubility or folding. This property should expand the choices of cellular compartments in which tagged antibodies can be functionally expressed. Furthermore, as the GFP11 tag is located at the opposite end of the binding interface, antigen-binding activity is preserved even when interacting with the complementary GFP1-10 fragment, as shown in figure 2.

A first series of experiments was carried out to investigate the possibility of fusing the GFP11 peptide to the C terminus of scFvs. We constructed a vector based on our pEP series [24]. This contained the SV5, GFP11 and His6 tags fused to the C terminus of the scFv, with restriction sites (*Bss*HII and *Nhe*I) compatible with our phage display vector, pDan5 [27], allowing rapid transfer following antibody selection by phage display (figure 1). The levels of scFv-GFP11 recovered after expression is comparable to the normal recovery of the same scFv-D1.3 without the GFP11 tag. The complementation between scFv-D1.3-GFP11 and GFP1-10 completely restores GFP fluorescence, conferring a highly stable fluorescence to the scFv, without interfering with the antigen binding activity of the single chain, as shown by the ELISA (figure 2) and FLISA experiments (figure 5). Comparing FLISA (figure 5) and ELISA (figure 2) carried out similarly, shows that ELISA is approximately twice as sensitive, able to detect down to ∼2.5 nM, while FLISA has a limit of detection of ∼5 nM). Once complementation has been carried out, detection using FLISA and GFP complementation does not require additional secondary reagents. Furthermore, as fluorescence is quantitative, it does not require protein purification, and providing the GFP1-10 is in molar excess, can be used to directly determine the amount of a GFP11 tagged protein in a crude mixture, as shown in figures 4. We were also able to show that GFP complementation can be used effectively in yeast display to label displayed scFvs, as an alternative to secondary antibodies. Furthermore, scFvs labeled with complemented GFP can be used in FLISAs (figure 5) and multiplexed flow cytometry (figure 6) to determine the approximate affinity of scFv of either purified (figure 7a) or crude (figure 7b) scFv preparations with similar levels of accuracy. This is likely to be of enormous utility in the high throughput selection and characterization of antibodies against large numbers of targets, where the ability to determine affinity rank without protein purification will increase the level of characterization that can be carried out. The use of GFP complementation to label scFvs has three advantages over the use of other methods: first each scFv is labeled with a single GFP molecule, second, the interaction between GFP11 and GFP1-10 is essentially irreversible under physiological conditions [19], and finally background due to fluorescent secondary reagents is eliminated as fluorescence is only generated upon complementation, although background due to adventitious by the scFv cannot be avoided.

Together these results show the great potential utility of GFP complementation in the use of scFvs in high throughput applications, including quantification, FLISA, multiplexed flow cytometry, affinity determination and yeast display.
